# Pollenless endeavour: loss of a glyoxal/galactose oxidase causes male sterility in rice

**DOI:** 10.1093/plphys/kiag730

**Published:** 2026-10-03

**Authors:** Gunjan Sharma

**Affiliations:** Assistant Features Editor, Plant Physiology, American Society of Plant Biologists; School of Biosciences, University of Birmingham, Edgbaston B15 2TT, United Kingdom

Seed plants reproduce through sexual reproduction by the male and female gametes produced in pollens and ovules, respectively. Viable pollens, vital for successful fertilization, are surrounded by an outer protective layer known as exine. The exine is deposited by the tapetum, an innermost somatic layer in the anthers in direct contact with developing pollens. The tapetum secretes nutrients such as sporopollenin, proteins, lipids, and enzymes through subsequent events including the programmed cell death (PCD) of specific target cells (Zhang et al. 2011; Walbot and Egger 2016). Timely degradation of the tapetum is critical for exine formation in developing pollens.

Tapetum degradation is a complex process orchestrated by a cascade of multiple transcription factors (Ortolan et al. 2024). Mutation of any of these factors causes delayed tapetum degradation, triggering the formation of nonviable pollen with defective walls, followed by pollen degeneration (Ortolan et al. 2024). Previous studies have shown that reactive oxygen species (ROS) produced from the NADPH oxidase RbohE trigger the degradation of tapetum (Yan et al. 2020). In addition to RbohE, a class of enzymes called glyoxal oxidases (GLOX) also produce H_2_O_2_ by the oxidation of aldehydes to carboxylic acids. In Arabidopsis, the *GLOX* gene shows induced expression during tapetum degradation, suggesting a role for GLOX-catalyzed H_2_O_2_ production during pollen development (Phan et al. 2011). However, the exact role of GLOX activity in rice pollen development is largely unexplored.

In this issue of *Plant Physiology*, **Liu et al. (2026)** revealed a previously unrecognized mechanism involving GLOX-mediated H_2_O_2_ production in tapetum degradation in rice. Forward genetic screening of a rice mutant library revealed a completely sterile mutant with pale shrunken anthers. The authors identified a point mutation in the rice *GLOX5* gene. OsGLOX5 contains glyoxal oxidase- and galactose oxidase-like domains, and the point mutation in *osglox5* is present in the glyoxal-like domain. The *osglox5* mutant pollen exhibited reduced chitin and sporopollenin content in the inner intine and outer exine layer, respectively, suggesting a defect in the formation of these layers. Further analysis revealed that the mutant anthers had delayed tapetum degradation. Complementation of *osglox5* rescued the pollen defects and male sterility, and removal of expression by independently knocking out *OsGLOX5* via CRISPR replicated the mutant phenotype, confirming *OsGLOX5* as the target gene controlling male sterility.

OsGLOX5 is an endoplasmic reticulum–localized protein expressed in the spikelets in the tapetum and microspores, suggesting a role in microspore development. The *osglox5* mutant plants accumulated less H_2_O_2_ than wild-type (WT), and the mutant form of OsGLOX5 showed reduced glyoxal- and galactose oxidase activity during in vitro enzyme assays, suggesting that GLOX5 effectively produces H_2_O_2_ in anthers. Furthermore, reduced levels of H_2_O_2_ in the *osglox5* mutant plants correlated with delayed tapetum degradation, suggesting that the activity of OsGLOX5 is essential for a timely degradation of the tapetum producing viable pollens.

Since *osglox5* mutants had a delayed tapetum degradation phenotype, the authors assessed the effect of known transcription factors facilitating timely degradation of tapetum and pollen development. *OsGLOX5* expression was downregulated in several mutants, and one transcription factor, OsMBY103 was shown by a dual-luciferase assay and chromatin immunoprecipitation (ChIP) to bind to the *OsGLOX5* promoter. Analysis of *osmyb103* mutants revealed loss of pollen viability and a delayed tapetum degradation along with lower H_2_O_2_ production, as seen in the *osglox5* mutants. Collectively, these findings suggest that OsMBY103 is an upstream regulator of *OsGLOX5* expression.

In conclusion, OsGLOX5 is a tapetum-expressed protein exhibiting both glyoxal- and galactose enzymatic activity that produces H_2_O_2_ which promotes tapetum degradation and sporopollenin deposition. Expression of *OsGLOX5* is induced by the direct binding of OsMYB103. A lower *OsGLOX5* expression in the *osglox5* mutants disrupts H_2_O_2_ levels, leading to slower degeneration of the tapetum and the formation of nonviable or no pollens, leading to male sterility (Fig. 1).

**Figure 1:**
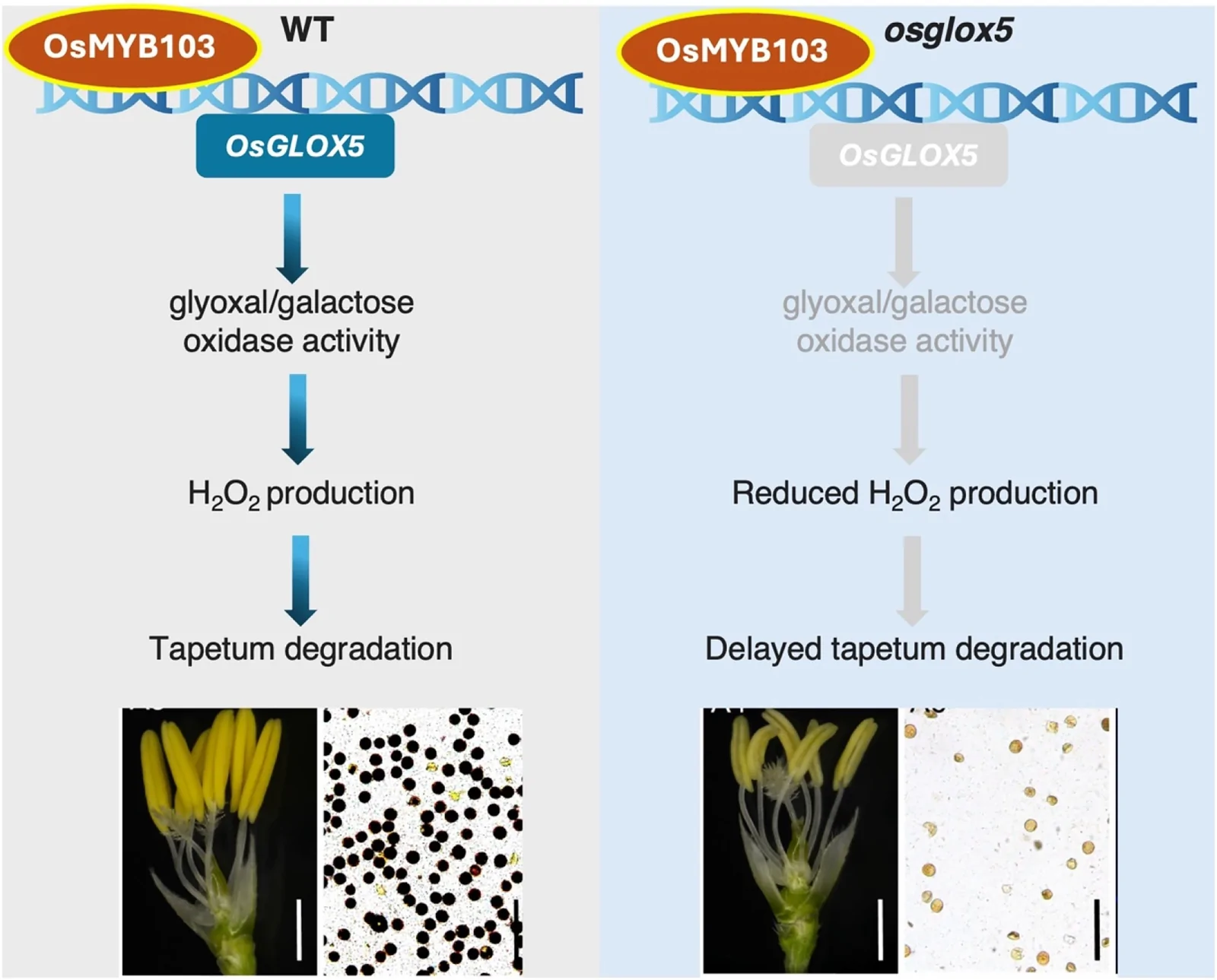
The OsMYB103-OsGLOX5 module regulates pollen development in rice. Development of viable pollen requires OsMYB103 binding to the *OsGLOX5* promotor. OsGLOX5 catalyzes the production of H_2_O_2_ essential for the timely degradation of the tapetum, which nourishes the developing pollen (WT). The absence of OsGLOX5 leads to a decreased H_2_O_2_ production delaying the tapetum degradation and forming nonviable pollen and male sterility. Gray colored arrows and text depict a decrease in expression and activity (adapted and modified from Liu et al. 2026).

Targeted early disruption of tapetal cells for generating male sterile lines with defective pollens is a widely used efficient breeding strategy for producing hybrid seeds, avoiding the need for manual anther removal (Marchant and Walbot 2022). The present study has enriched the toolkit for generating male sterile lines. Delaying the tapetum degradation through altering OsMYB103 or OsGLOX5 could be valuable in rice breeding programs. Further investigations in different rice varieties would provide the robustness for wide implantation.

## Related articles published in *Plant Physiology*

Lu et al. (2024) identified MORE FLORET 1 controlling pollen development in diploid and tetraploid rice with a potential of assisting breeding programs for generating polyploid male sterile lines for producing hybrid rice.

Wang et al. (2026) identified a peroxidase TaPOD8 regulating male sterility in wheat by maintaining ROS production and protection against DNA damage in wheat reproductive cells.

## Data Availability

No new data were generated or analyzed for this article.
